# The Evolution of Finger Counting between Kindergarten and Grade 2

**DOI:** 10.3390/children9020132

**Published:** 2022-01-20

**Authors:** Céline Poletti, Marie Krenger, Justine Dupont-Boime, Catherine Thevenot

**Affiliations:** 1 Institute of Psychology, University of Lausanne, 1015 Lausanne, Switzerland; celine.poletti@unil.ch (C.P.); marie.krenger@unil.ch (M.K.); 2 Psychology Section, University of Geneva, 1205 Geneva, Switzerland; justine.dupontboime@gmail.com

**Keywords:** numerical cognition, arithmetic, calculation, finger use, individual differences

## Abstract

In this longitudinal study, we aimed at determining whether children who efficiently use finger counting are more likely to develop internalized arithmetic strategies than children who are less efficient. More precisely, we analyzed the behavior of 24 kindergarteners aged between 5 and 6 years who used their fingers to solve addition problems, and we were interested in determining the evolution of their finger counting strategies towards mental strategies after 2 years (Grade 2). Our results show that kindergarteners who were the most proficient in calculating on fingers were the more likely to have abandoned this strategy in Grade 2. This shows that the use of efficient finger counting strategies early during development optimizes the shift to mental strategies later on during school years. Moreover, children who still use their fingers to solve additions in Grade 2 present lower working memory capacities than children who had already abandoned this strategy.

## 1. Introduction

The question of whether children should be encouraged to use their fingers to solve calculations is often asked amongst teachers and practitioners [1]. Researchers in the domain of developmental psychology have recently addressed this question. Thus, we know that kindergarten children who use their fingers to solve additions are more cognitively efficient than children who do not. More precisely, they present higher working memory capacities [2] and higher numerical performance [3], noticeably in arithmetic tasks [2,4,5,6]. These results could be surprising, as counting on fingers is sometimes viewed as an immature behavior used by children who do not master a better strategy, and need their fingers to release working memory resources [7,8,9,10]. The results of Dupont-Boime and Thevenot suggest, on the contrary, that cognitive resources are needed to implement an efficient finger counting strategy, and that unfortunately, children who are cognitively limited cannot invent and apply it [2]. In support of this conclusion, it has also been shown that when children with high and lower cognitive resources use their fingers to solve a problem, the former are more likely to use more sophisticated counting strategies such as counting from a given addend rather than counting all from a given addend [2,10]. As noted by Baroody, counting on fingers can be quite a sophisticated invention, and it is therefore understandable that children with limited cognitive resources present delays and difficulties in implementing this strategy [11].

Not surprisingly, the result that children who use their fingers in an arithmetic task are more efficient than children who do not is specific to young children. At a later age, in fact as soon as the beginning of Grade 1, the positive correlation between performance and finger counting starts to decline [4], until it disappears in Grade 2 [4,12], and even reverses at the end of Grade 2 [4]. At a more theoretical level, these results reflect the commonly described development of arithmetic strategies consisting in a shift from overt, slow and effortful strategies to internalized and faster ones [13,14,15,16]. In children who develop optimally, mental strategies should progressively become the dominant strategy. In fact, as noticeably suggested by Baroody [11], practicing finger counting strategies where both operands are represented on fingers (e.g., 4 + 3 = 1, 2, 3, 4 on one hand; 1, 2, 3 on the other and counting the 7 fingers) allows children to progressively shift towards a strategy where the second operand is enumerated rather than counted (i.e., 4 + 3 = 1, 2, 3, 4 on one hand and enumerate of 5, 6, and 7 on the other). This transition in finger counting strategies would eventually allow children to represent only one operand on fingers and finally to solve the problem completely mentally. In short, practice and proficiency in finger counting could scaffold and optimize the development of internalized strategies. Stated differently, practicing arithmetic problem solving on fingers would progressively allow children to construct abstract representations. This proposition echoes the one by Andres, Di Luca & Pesenti [17], who consider fingers as possible means to shift from non-symbolic to symbolic numerical representations. The idea that efficiency in finger counting leads to efficiency in manipulating abstract numerical representations during addition tasks will be examined in the present paper. 

To this aim, we adopted a longitudinal approach where we tested children in Kindergarten at the age of 5-to-6 years, and then in Grade 2 at the age of 7-to-8 years. This approach allowed us to examine the evolution of finger counting strategies, and to establish children ‘s profiles. We focused our attention on 24 children who used their fingers in Kindergarten. We were interested not only in determining the proportion of children who still used finger counting two years later in Grade 2 (i.e., referred to as “keepers” in the remaining of the text), but also in the profile of children who had already abandoned this finger counting strategy (i.e., referred to as “stoppers”). More precisely, we aimed at determining whether keepers and stoppers in Grade 2 could be differentiated on the basis of their performance in Kindergarten. 

We hypothesize that children who do not use their fingers to count any longer in Grade 2 correspond to children who used their fingers the most efficiently in Kindergarten. Moreover, while as already explained above, young children who use their fingers to solve calculations at the age of 5-to-6 years present the highest working memory resources, we will verify, in accordance with Geary et al. [10], that this relation is no longer significant at an older age. This would confirm previous assumptions and conclusions of the literature that a limitation in working memory capacities is associated with a too-persistent reliance on finger counting strategies during development [9,10,18].

## 2. Material and Methods

### 2.1. Participants

Seventy-six French-speaking Swiss kindergarteners from different socioeconomic backgrounds from four classrooms in several schools of Geneva took part in the experiment. The homogeneity of teaching methods across schools was ensured by an educational curriculum program applied in the whole French part of Switzerland (i.e., PER, for Plan d’Etude Romand). In this PER, finger counting is neither encouraged nor discouraged. 

In order to base our conclusions on reliable data, only the data of children with their hands visible at least for 50% of the trials at each of the two testing points was kept for our analyses. For example, when children’s hands were under the table, we could not determine whether they counted on their fingers, and we had therefore to consider the trial as invalid. The fact that finger counting was not used throughout the experiment was obvious for only 2 children. This does not mean that other children did not use their fingers to count, but that their hands could have been out of the experimenter’s sight. Therefore, we decided to focus our analyses on 24 (15 girls) children who unambiguously used their fingers. These children were tested for the first time between the age of 5 and 6 years in February, during the second semester of their second year of schooling (Kindergarten, 2P in Switzerland: M = 72 months, SD = 4 months, range from 66 to 77 months). They were tested again between the age of 7 and 8 years, during the second semester of their fourth year of schooling in June (Grade 2, 4P in Switzerland, M = 100 months, SD = 4 months; Range from 94 to 105 months). 

None of these children presented developmental disorders or disabilities. For all children, written informed consent was obtained from the parents before testing, and the study was approved by the local Ethics Committee.

### 2.2. Material and Procedure

At both testing points, children were assessed individually for about 30 min. They performed an addition task and were evaluated on their working memory capacities.

#### 2.2.1. The Addition Task

Children were asked to solve 12 single-digit additions presented vertically on paper cards. The additions were constructed with addends from 1 to 9 and sums from 3 to 16. Half of them were small additions with a sum smaller than 10, and the other half were larger additions with a sum larger than 10 (see Appendix A). Tie-problems and additions with a sum equal to 10 were excluded, as they are known to be easily solved by memory retrieval and are therefore less likely to be solved by finger counting [14,19,20]. Additions involving a 0 were also excluded, because they cannot be solved using fingers.

Problems were answered using stickers because we did not want children to hold a pen in their hand during the solving process, which could have prevented them from using their fingers during the task. Twenty-four stickers with Arabic digits corresponding to the potential problem answers were placed on the table, and children had to select the sticker corresponding to the solution of the addition. Each answer sticker was presented twice on the table, so that children could not deduce the correct responses by the end of the task. All additions on paper cards and potential answers on stickers were presented altogether on the table, and children could solve the problems in the order they wanted. No instruction was given to children regarding the use of fingers.

Children’s behaviors were videotaped using a discrete camera-pen. The videos were watched and coded after recordings, and for each problem, it was noted whether children used their fingers or not. The session started after one training trial corresponding to a problem that was not re-used during the experimental phase. The task lasted between 2 and 7 min, depending on the speed and the motivation of the children.

#### 2.2.2. The Working Memory Task

Working memory was assessed in kindergarteners with the backward digit span of the WISC-IV, wherein children are asked to repeat a sequence of digits (e.g., 2-9-5) in reverse order (e.g., 5-9-2). Digits were uttered verbally with a rate of approximately 1 per second, and children had no time limit to repeat them. The material of the backward digit span is composed of 8 sequences ranging in length from 2 to 8 digits. Each sequence consists of 2 trials where the digits vary from 1 to 9. The task ends when the child fails 2 consecutive trials of a same length. This length, or in other words the maximum amount of digits that a child can repeat backwards, corresponds to his or her working memory span.

## 3. Results

As already stated, we studied 24 kindergarteners who used their fingers at least once to solve a problem in an addition task. At the second point of testing, 2 years later, 11 children (6 girls) still used their fingers to solve the problems, while the other 13 (9 girls) had already abandoned this strategy. The following analyses will be first conducted on the percentage of finger counting in the addition task by examining this variable in light of the testing point (i.e., Kindergarten and Grade 2) and the size of problems (i.e., small and large), and then by examining this variable in Kindergarten only, depending on whether children continued or stopped using their fingers in Grade 2 (i.e., “keepers” and “stoppers”). Second, we will analyze the percentage of correct responses for keepers and stoppers in Kindergarten and Grade 2 for small and large problems. Finally, we will examine whether keepers and stoppers can be differentiated based on working memory capacities.

### 3.1. Percentage of Finger Counting

**Figure 1:**
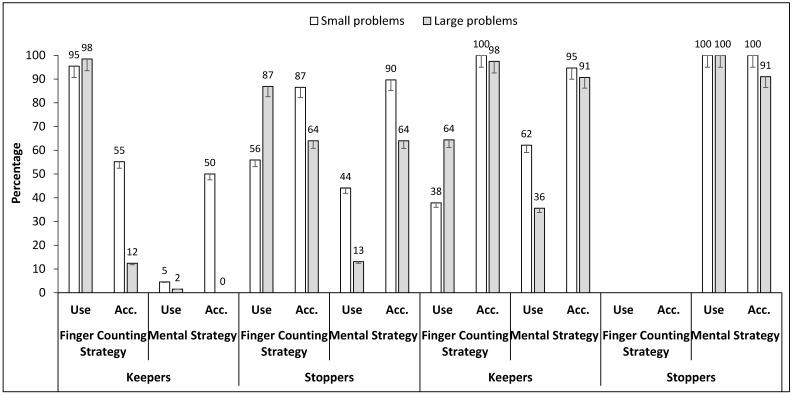
Percentage of finger and mental counting strategies and accuracy in Kindergarten and in Grade 2 in keepers and stoppers. Acc. for Accuracy = Correct answers.

A 2 (Testing point: Kindergarten, Grade 2) × 2 (Problem size: small, large problems) ANOVA, with both factors as repeated measures was performed on the percentage of finger counting (see Figure 1 for the overall pattern of result and Table 1 for a description of the data at an individual level). 

The results showed that children more often used their fingers in Kindergarten than in Grade 2 (83% of the problems vs. 23%, *F*(1, 23) = 102.04, *n²p =* 0.82, *p* < 0.001). They also used their fingers more often for large than for small problems (61% vs. 46%, *F*(1, 23) = 22.32, *n²p =* 0.49, *p* < 0.001). The Testing point x Problem size interaction was not significant *F* < 1, *p* = 502) showing that the effect of Problem size was not larger in Kindergarten (+18% for large problems) than in Grade 2 (+12%).

The percentage of finger counting was then analyzed in Kindergarten depending on children’s behavior in Grade 2 (Table 1). A 2 (Group: keepers, stoppers) × 2 (Problem size: small, large problems) ANOVA, with the first factor as a between measure and the second as a repeated measure was performed on the percentage of finger counting in Kindergarten (see Figure 1). The results revealed a main effect of Group showing that, in Kindergarten, stoppers already used their fingers less often than keepers (71% vs. 97%, *F*(1, 22) = 9.84, *n²p =* 0.31, *p* = 0.005). There was also an effect of Problem size showing that the percentage of finger counting was higher for larger problems (93% vs. 76%, *F*(1, 22) = 12.21, *n²p =* 0.36, *p =* 0.002). The Group x Problem size interaction was significant (*F*(1, 22) = 8.25, *n²p =* 0.27, *p =* 0.009) showing that the problem size effect was higher (+31% for large problems, *F* = 22.11, *p* < 0.001) in stoppers than in keepers, where it was not significant (3%, *F* < 1, *p* = 0.677).

In Grade 2, the analysis concerning the percentage of finger counting could not be conducted in stoppers who, by definition, did not use their fingers any longer. The results showed that keepers more often used their fingers for large than for smaller problems (64% vs. 38%, *F*(1, 22) = 6.54, *n²p =* 0.40, *p* = 0.028).

### 3.2. Percentage of Correct Responses in the Addition Task

A 2 (Group: keepers, stoppers) × 2 (Testing point: Kindergarten, Grade 2) × 2 (Problem size: small, large problems) ANOVA, with the first factor as a between-measure and the two last as repeated measures was performed on the percentage of correct responses (see Figure 1 and Table 1).

The percentage of correctly solved problems was higher in Grade 2 than in Kindergarten (97% vs. 55%, *F*(1, 22) = 74.71, *n²p* = 0.77, *p* < 0.001) and higher for small than larger problems (85% vs. 66%, *F*(1, 22) = 39.46, *n²p* = 0.64, *p* < 0.001). The analysis also revealed a main effect of Group showing that stoppers were more accurate than keepers (85% vs. 66%), *F*(1, 22) = 13.63, *n²p* = 0.38, *p* = 0.001, but the Group × Testing point interaction (*F*(1, 22) = 20.68, *n²p* = 0.49, *p* < 0.001) revealed that it was true only in Kindergarten (75% vs. 34%, respectively in stoppers and keepers, *F* = 17.93, *p* < 0.001). In Grade 2, stoppers and keepers were equally accurate with quasi-ceiling performance in the two groups (95% vs. 99%, *F* = 1.82, *p* = 0.190). Testing point also interacted with Problem size (*F*(1, 22) = 13.48, *n²p* = 0.38, *p* = 0.001), showing that the problem size effect was higher (+33% for small problems, *F* = 26.97, *p* < 0.001) in Kindergarten than in Grade 2 (+6%, *F* = 5.54, *p* = 0.028). Finally, the Group × Testing point x Problem size interaction was marginally significant (*F*(1, 22) = 3.33, *n²p* = 0.20, *p* < 0.082), showing that the problem size effect in Kindergarten was higher in keepers (+43% for small problems, *F* = 21.29, *p* < 0.001) than in stoppers (+23%, *F* = 7.05, *p* = 0.014), whereas in Grade 2, the problem size effect was still present in stoppers (+9% for small problems, *F* = 7.49, *p* = 0.012), but not in keepers (+2%, *F* < 1).

### 3.3. Finger Counting and Working Memory Spans

One child (ID = 86 in Table 1) was excluded from this analysis, as her score in working memory was not available. Children’s mean span in Kindergarten was 2.65 (SD = 0.71), but children who will become stoppers in Grade 2 had a higher memory span (M = 3.09, SD = 0.67) than keepers (M = 2.18, SD = 0.41), *t* (21) = −3.87, *p* < 0.001, *d* = −1.614). This result was confirmed by the creation of a dichotomous variable. Children with a memory span between 1 and 2 were categorized as “low-span”, and children with a memory span between 3 and 4 were categorized as “high-span”. Based on this classification, the results showed that 10 out of 12 high span children were stoppers whereas 10 out of 11 low span children were keepers. A chi-square test confirmed that being a stopper or a keeper was not independent of working memory spans, (*χ^2^*(1, *N* = 23) = 9.76, *p* < 0.01).

## 4. Discussion

In this study, we were interested in investigating the role of finger counting on addition performance in early schooling. More precisely, we aimed at determining whether children who use their fingers efficiently to count in Kindergarten are those children who abandon them earlier during development for this activity. More generally and going further, we aimed at determining whether children who still used their fingers or not in Grade 2 could already be differentiated in Kindergarten based on their behavior and performance. We hypothesized that practice and proficiency in finger calculation optimize the development of internalized strategies. Our research confirms our hypotheses.

We focused our attention on 24 kindergarteners aged between 5 and 6 years who used their fingers to solve addition problems. More precisely, they solve 84% of simple addition problems using their fingers, and reached 55% of correct responses. When the remaining 16% of problems were considered, the percentage of correct answer was 51%. As we will see below, these results were strongly modulated by the characteristics of the problems that we studied (i.e., small or larger) and the characteristics of children. Two years later, in Grade 2, only 26% of the problems were still solved using finger counting. In fact, 13 children had completely stopped using their fingers and were qualified as stoppers. At this second testing point, when only children who still used their fingers were considered (i.e., keepers), half of the problems were still solved using fingers.

One strength of our longitudinal approach is that we could examine the characteristics of keepers and stoppers back in Kindergarten. This revealed that two years before Grade 2, stoppers already used their fingers less often (71%) than keepers (97%). Therefore, children who still used their fingers in Grade 2 solved almost all the problems with their fingers in Kindergarten. Using this strategy, this group of kindergarteners solved only one third of problems correctly, and only 12% of large problems. For the anecdotal part of problems that were solved mentally by this group of children, the percentage of correct answers was 25% (with 0% for large problems). These children were undoubtedly less efficient than children who had already stopped using their fingers in Grade 2. Indeed, the stoppers solved 75% of the problems correctly with their fingers in Kindergarten, and 77% of the problems correctly when using a mental strategy. The higher efficiency of stoppers over keepers was also apparent in their higher working memory capacity. In fact, the group of stoppers was constituted almost exclusively of children with the highest working memory span, except for two children. Conversely, the group of keepers was almost exclusively constituted of children with the lowest span. These results corroborate previous conclusions of the literature, showing that an over-reliance on finger counting is a characteristic of children presenting difficulties in arithmetic, and also suffering from working memory limitations [9,18,21]. On a more general level concerning the use of finger strategies by children with high and lower working memory capacities, it has to be noted that, in support of the results by Geary and Brown [22], more gifted children use more diverse strategies, and use them more adaptively than less gifted children.

This discussion about finger counting strategies cannot be conducted independently of the characteristics of the problems solved by children. At a general level and in replication to all previous studies in young children [14], we obtained a problem size effect showing that larger problems were more often failed than smaller ones. However, this was no longer true in Grade 2 for keepers, due to the ceiling effect. Interestingly, the residual size effect in stoppers was due to the relatively low percentage of correct responses for large problems solved mentally (91% compared with 100% for smaller problems). This percentage of 91% was lower than the 98% of success in keepers when they used their fingers. This suggests that stoppers could have benefited from keeping the finger strategy to solve some large problems a little bit longer during development.

To sum up, compared to children who have already abandoned the finger counting strategy in Grade 2, kindergarteners who still use this strategy two years later suffer from working memory limitations, and generally present poor solving performance in addition whatever the strategy they use (i.e., finger or mental). This pessimistic description of keepers could be balanced by the fact that they reach the same performance as stoppers in Grade 2. However, it is not surprising that 8-year-old children without learning disabilities do not fail in solving very simple addition problems with a sum up to 16. In fact, efficiency is not always a matter of accuracy but the time needed to solve a problem can also be very detrimental for achieving arithmetic fluency, as long solution times decrease either the probability of associations in memory [23,24] or the probability of automatization of counting procedures [25]. As a matter of fact, counting on fingers take longer than relying on internalized strategies [14], and keeping this strategy over too long a developmental period could therefore be problematic. In future studies, children’s solution times when they use their fingers or not should be systematically measured.

Our results show that children who use their fingers to solve addition problems in Kindergarten and still keep using them at least until Grade 2 are not so efficient compared to children who have already abandoned their fingers in Grade 2, which could seem at odds with the conclusion of the literature that kindergarteners who use their fingers are more efficient than those who do not [2,4]. Indeed, from our current results, it could be concluded that children who use their fingers over a “too”-long period of time are those children with the lowest arithmetical skills. This is not necessarily the case, however, as we have not compared them with children who had never used their fingers to calculate. It could perfectly be that this last category of children presents even more difficulties than children who have not abandoned yet the finger counting strategy in Grade 2. As a matter of fact, it has repeatedly been shown that kindergarteners who do not use their fingers at all in arithmetic tasks are less efficient, and present lower cognitive abilities than children who use the finger counting strategy [2,4,5,26]. Therefore, we show here that children who use their finger early during development and abandon them quickly to solve problem are more efficient than children who keep using them; this does not mean that in Kindergarten finger users are less efficient than children who do not use their fingers at all. Again, this last category of children was not part of our study.

Our conclusion that the winning trajectory for the development of good arithmetic abilities seems to be to use finger counting early, but to abandon this strategy quickly during the course of development is original. Indeed, it was already known that the use of fingers in Grade 2 was not associated with good arithmetic performance [4,12], whereas this correlation was significant in Kindergarten [2,4]. However, it was not known whether efficient children in Grade 2 had already abandoned this strategy or had never used it, or whether less efficient children were using the finger strategy for several years or just discovered it. Therefore, we think that our longitudinal approach wherein children’s individual trajectories are examined is promising. As already evoked earlier, this approach should be used at a fine-grained level with several assessments of children during a specific school level and across consecutive and numerous school levels. Moreover, several aspects of numerical cognition such as enumeration, numerical comparison or arithmetic in general could be investigated in order to determine whether adaptive and early finger counting is associated and even, as suggested by several researchers, boost the development of good numerical abilities [17,27,28,29,30]. The results of these complementary studies could reinforce the idea that could be derived from our present results, according to which, at the very least, finger counting should not be discouraged in schools.

Before concluding, we have to acknowledge that the promising and original results that we present in this paper should be considered as a basis for further research, and not as definite results resolving or closing research questions. Indeed, individual observations of children’s behaviors over a sufficient period of time are very demanding, and we have paid this cost in the size of our children sample, which is relatively small for group analyses. Future studies will have to observe more children in order to conduct more reliable analyses. Additionally, a closer look at the specific finger counting strategies that children used (i.e., ALL, Min.) would have been precious. This is an approach that we have started to follow, and the results are also very promising, but again, the small size of our sample did not allow us to divide our groups further based on the strategies used. This is also a line of research that will need to be pursued in future studies. Finally, if further investigation about the relation between working memory and finger counting should be conducted, using more than one working memory task and using tasks that do not involve numerical digits is recommended. Indeed, the working memory task was used to measure abilities that are independent from numerical skills, and it could be considered that children who are already not so at ease with numbers are not very motivated in a task where numbers are involved.

## Figures and Tables

**Table 1:** Percentage of finger counting and mental strategies and accuracy in Kindergarten (left half of the Table) and in Grade 2 (right half of the Table) in keepers and stoppers. Means are reported in bold.

				Kindergarten								Grade 2							
				Percentage of Finger Counting		Accuracy with Finger Counting		Percentage of Mental Counting		Accuracy with Mental Counting		Percentage of Finger Counting		Accuracy with Fingers Counting		Percentage of Mental Counting		Accuracy with Mental Counting	
ID	Gender	Group	Memory Span	Small	Large	Small	Large	Small	Large	Small	Large	Small	Large	Small	Large	Small	Large	Small	Large
7	Girl	Keepers	2	100	100	25	0	0	0	-	-	50	100	100	75	50	0	100	-
24	Boy	Keepers	2	100	100	100	33	0	0	-	-	0	83	-	100	100	17	100	100
26	Girl	Keepers	2	100	100	0	0	0	0	-	-	0	17	-	100	100	83	75	60
30	Boy	Keepers	2	100	100	60	0	0	0	-	-	0	67	-	100	100	33	100	100
55	Girl	Keepers	3	100	100	20	0	0	0	-	-	67	33	100	100	33	67	100	100
61	Boy	Keepers	2	75	100	67	0	25	0	0	-	20	33	100	100	80	67	100	75
66	Boy	Keepers	2	100	100	33	0	0	0	-	-	40	75	100	100	60	25	67	100
81	Girl	Keepers	3	100	100	100	0	0	0	-	-	80	100	100	100	20	0	100	-
83	Girl	Keepers	2	100	100	60	0	0	0	-	-	60	100	100	100	40	0	100	-
84	Boy	Keepers	2	75	83	67	20	25	17	100	0	20	0	100	-	80	100	100	100
85	Girl	Keepers	2	100	100	75	83	0	0	-	-	80	100	100	100	20	0	100	-
**Mean**			**2**	**95**	**98**	**55**	**12**	**5**	**2**	**50**	**0**	**38**	**64**	**100**	**98**	**62**	**36**	**95**	**91**
33	Girl	Stoppers	2	100	100	50	0	0	0	-	-	-	-	-	-	-	-	100	100
35	Boy	Stoppers	3	100	100	100	80	0	0	-	-	-	-	-	-	-	-	100	100
36	Boy	Stoppers	3	67	100	100	60	33	0	100	-	-	-	-	-	-	-	100	100
40	Girl	Stoppers	3	50	100	100	100	50	0	100	-	-	-	-	-	-	-	100	83
44	Girl	Stoppers	4	25	80	100	75	75	20	67	100	-	-	-	-	-	-	100	100
45	Girl	Stoppers	4	80	100	75	60	20	0	100	-	-	-	-	-	-	-	100	100
56	Girl	Stoppers	2	60	67	67	50	40	33	50	-	-	-	-	-	-	-	100	83
65	Boy	Stoppers	3	80	83	100	100	20	17	100	100	-	-	-	-	-	-	100	100
67	Girl	Stoppers	4	0	100	-	0	100	0	100	-	-	-	-	-	-	-	100	100
68	Girl	Stoppers	3	25	83	100	100	75	17	100	100	-	-	-	-	-	-	100	83
75	Boy	Stoppers	3	40	100	100	40	60	0	100	-	-	-	-	-	-	-	100	83
82	Girl	Stoppers	3	100	100	60	67	0	0	-	-	-	-	-	-	-	-	100	50
86	Girl	Stoppers	NA	0	17	-	100	38	28	80	20	-	-	-	-	-	-	100	100
**Mean**			**3**	**56**	**87**	**87**	**64**	**44**	**13**	**90**	**64**	**-**	**-**	**-**	**-**	**-**	**-**	**100**	**91**

## Data Availability

The sets of data described and analyzed in the present paper can be found here: https://osf.io/uy85h/?view_only=7977e2c36f1b4a0685ce28bbcdf7a64f (accessed on 26 December 2021).

## Appendix A

**Table A1:** List of problems used in the addition task.

6 Additions: Sum < 10	6 Additions: Sum > 10
2 + 1	7 + 4
2 + 3	8 + 4
4 + 2	6 + 7
1 + 6	8 + 6
3 + 5	7 + 8
3 + 6	9 + 7
